# A Fluidic Interface with High Flow Uniformity for Reusable Large Area Resonant Biosensors

**DOI:** 10.3390/mi8100308

**Published:** 2017-10-14

**Authors:** Charles-Louis Azzopardi, Vivien Lacour, Jean-François Manceau, Magali Barthès, Dimitri Bonnet, Franck Chollet, Thérèse Leblois

**Affiliations:** 1 FEMTO-ST Institute, Univ. Bourgogne Franche-Comté, CNRS, 15B avenue des Montboucons, 25030 Besançon, CEDEX, France; cl.azzopardi@femto-st.fr (C.-L.A.); vivien.lacour@femto-st.fr (V.L.); jfmanceau@femto-st.fr (J.-F.M.); magali.barthes@femto-st.fr (M.B.); dimitri.bonnet@femto-st.fr (D.B.); franck.chollet@femto-st.fr (F.C.); 2 Institute for Interdisciplinary Innovations in Technology (3IT), Faculty of Engineering, Université de Sherbrooke, 3000 Boulevard de l’Université, Sherbrooke, QC J1K OA5, Canada

**Keywords:** microengineering, planar flow, fluidic interface, micro-machining, biosensor

## Abstract

Resonant biosensors are known for their high accuracy and high level of miniaturization. However, their fabrication costs prevent them from being used as disposable sensors and their effective commercial success will depend on their ability to be reused repeatedly. Accordingly, all the parts of the sensor in contact with the fluid need to tolerate the regenerative process which uses different chemicals (H_3_PO_4_, H_2_SO_4_ based baths) without degrading the characteristics of the sensor. In this paper, we propose a fluidic interface that can meet these requirements, and control the liquid flow uniformity at the surface of the vibrating area. We study different inlet and outlet channel configurations, estimating their performance using numerical simulations based on finite element method (FEM). The interfaces were fabricated using wet chemical etching on Si, which has all the desirable characteristics for a reusable biosensor circuit. Using a glass cover, we could observe the circulation of liquid near the active surface, and by using micro-particle image velocimetry (μPIV) on large surface area we could verify experimentally the effectiveness of the different designs and compare with simulation results.

## 1. Introduction

**Figure 1:**
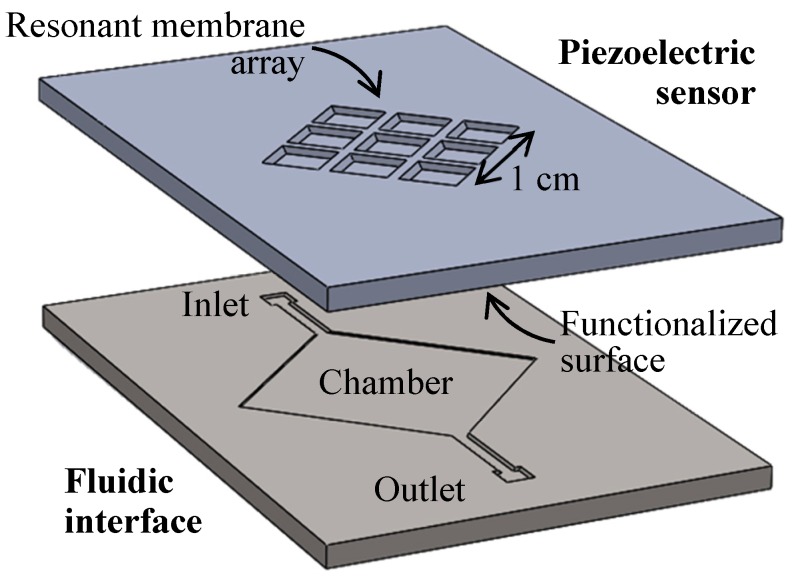
Exploded view of an acoustic biomedical microelectromechanical systems (BioMEMS) sensor, based on the assembly of an array of functionalized piezoelectric membranes with a microfluidic interface, for real time monitoring of specific molecules capture.

Piezoelectric devices, such as bulk acoustic wave (BAW) or surface acoustic wave (SAW) devices, have started to be used successfully for biosensing purposes. In fact, they exhibit high sensitivity in mass [1,2], in viscous/viscoelastic changes [3,4], as well as in the detection of conformational changes of surface-bound biomolecules for better understanding of biomolecular interactions [5,6]. In the field of biosensors, the major strengths of the resonant piezoelectric devices are their high sensitivity and versatility and their capacity to yield wireless and portable devices of very small size. However, the high level of miniaturization brings its share of drawbacks. First, the microfabrication in a clean-room of the sensitive part with piezoelectric materials is relatively expensive. Moreover, it is hard to integrate in these materials the microchannels and chamber needed for the manipulation of fluids at such a small scale. To circumvent these two issues, we will describe a microfluidic interface that can be integrated within a reusable piezoelectric sensor, for bringing biofluids in contact with the sensitive surfaces (Figure 1).

This fluidic interface will essentially help control the flow rate over the functionalized surface to obtain a good balance between convective and diffusive flow to control the dynamic of the sensor [7,8]. But additionally, as we have a sensitive area of about 1 cm^2^ composed of an array of membranes, the interface will need to ensure that the flow is even over this large area to make sure each membrane captures a similar amount of bio-analyte. The interface should also be bio-compatible and not release any chemicals or particles during its use. It should exhibit a good chemical resistance to withstand different analytes and, more importantly, the chemical used for sensor regeneration in this reusable sensor. Finally, as the fluidic interface will be assembled with the membrane array, it should also match the mechanical properties of the piezoelectric materials used for their operation.

We find in the literature fluidic interfaces with a single inlet/single outlet configuration (with or without taper) but they inevitably lead to marked flow nonhomogeneity [8,9]. For improving this structure, manifolds have been used at the inlet and/or outlet to evenly spread the fluid at the chamber edge [10]. However, we find few systematic studies that try to optimize the flow uniformity, particularly in the case of large dimension chambers (1 cm^2^). Saias, et al. [8] proposed to improve compactness of the manifold structure to obtain uniform flow in a rectangular chamber. In their study, they do not discuss diamond shape chambers, although they recognize the superiority of rhombus architecture. Moreover, their experimental results only show the magnitude of the velocity along a single line but do not provide the full velocity vector field in the chamber. This last information could give a better view of the difference between simulation and experimental results. Recently, Pálovics, et al. [9] improved flow in a circular chamber filled with a porous material by developing a structure with 3 inlets and 3 outlets spaced by 30°. In their work, the optimization criteria was the residence time in the chamber and not the flow uniformity, as they were optimizing chemical reaction. In another work, Liao, et al. [10] compared two bifurcate tree-branch structures with four levels feeding a square 1 cm^2^ chamber. One of the structures had channels of constant width and the other used a biomimetic structure where between each level of the manifold the width of the channel is divided by two, following Murray’s law [11]. They showed that the biomimetic design increased photocatalytic reaction, but from simulation results explained that if the pressure in the chamber was kept to a lower level, it presented a less uniform velocity profile. Moreover, there was no direct experimental measurement of the flow velocity in the chamber, but they only reported the net photocatalysis results.

In this paper, our take at this issue is exposed in four parts. First, we present the design of the reusable biosensor and substantiate the fluidic interface’s structural choices by presenting simulation of the fluid flow in selected architectures. Then, we detail the reason behind the material choice and the fabrication process of the microfluidic circuits, focusing on diamond-shaped chambers. In the last experimental section, we propose a set-up for recording the full velocity field inside the chamber and eventually confront the experimental results with theory for discussion.

## 2. Design and Simulation

### 2.1. Fluidic Interface Characteristics

The global objective for the biosensor is at least to match the resolution of a standard SPR system that is placed somewhere near 0.1 nmol/L (or commonly described as 0.1 nM with M, named molar, representing the 1 mol/L concentration) [12,13]. The sensor (Figure 1) separates the fluidic and the sensitive parts [14], allowing one to optimize them independently, and particularly ensuring good compatibility between bio-interface grafting and etching [15,16]. The sensitive part of the sensor has been described elsewhere [14], but we will present the main point of its design to better understand the key parameters of the fluidic interface. The piezoelectric resonant sensor is built with GaAs, a piezoelectric material where the grafting process with thiolate is comparable to grafting on gold, for which many analytes are readily available. Biofunctionalization is performed on deoxidized GaAs surface and we verified that the grafting of molecules induces a shift in the resonant frequency, and that the thiolate functionalization can be regenerated [17]. For a resonating membrane with a side of about 3 mm and with a thickness of about 3 μm, the fundamental mode is at 557 MHz, giving a mass sensitivity of about 350 fg/Hz [14]. For increased reliability—and ultimately a multi-sensing approach—we use redundancy, and the sensor is based on a 3 × 3 array of identical resonant membranes facing the fluidic cavity, resulting in an overall sensing area of about 1 cm^2^.

For estimating the bio-sensing characteristics of the sensor, we will consider the capture of a typical protein of a mass of 100 kDa (thus weighting $m=1.6\times 10^{-19}$ g/molecule). With a frequency resolution of 1 Hz, the sensor has a resolution of 350 fg corresponding to the capture of about $2\times 10^{6}$ protein molecules. With the 3 × 3 mm^2^ sensor membrane and an estimated $10^{11}$ binding sites/cm^2^ we get about $10^{10}$ binding sites on each membrane that will result in a comfortable full-scale range of about 5000 times the sensor resolution.

The dynamic of the sensor is mostly controlled by the rate at which the bio-analyte reaches the functionalized surface. But, this transport issue is complicated because a depletion zone appears above the membrane when the bio-analyte binds to its functionalized surface [7]. The operating regime of the sensor needs to be estimated by comparing characteristic diffusion and convection times away from the sensing surface (with characteristic channel depth *H*) and along this surface (with characteristic length *L*). In both cases, this amounts to evaluating a Péclet number, respectively, ${\mathrm{Pe}}_{H}$ and ${\mathrm{Pe}}_{L}$:

(1) $${\mathrm{Pe}}_{H}=\frac{\bar{v}H}{D}\;{\mathrm{Pe}}_{L}=6\frac{\bar{v}L^{2}}{HD}.$$

In the case of a diffusivity of *D* = 50 μm^2^/s, corresponding to a typical protein of 100 kDa in water, a depth of the chamber of *H* = 80 μm and a length of the sensor of *L* = 3 mm, we get ${\mathrm{Pe}}_{H}=1.6\times 10^{6}\bar{v}$ and ${\mathrm{Pe}}_{L}=1.3\times 10^{10}\bar{v}$ where $\bar{v}$ is the average velocity of fluid in the chamber. They are both much larger than 1 for standard fluid velocity ($\bar{v}>10$ μm/s) in the chamber, meaning in the first case that the depletion zone is thinner than the chamber depth and in the second case that it is much shorter that the sensor membrane. The mass-transport flux of molecules to the sensor surface is then given by [7]

(2) $$J\approx Dc_{0}W\;\sqrt[{3}]{{\mathrm{Pe}}_{L}}$$

giving for a target concentration of biomolecules of *c*_0_ = 0.1 nM and a square sensor (*W* = *L* = 3 mm) a net bio-analyte flux of $J\approx 2.1\times 10^{7}\;\sqrt[{3}]{\bar{v}}$ molecules/s.

The transport of the bio-analyte needs to be much faster than its binding speed so that mass-transport doesn’t hamper the binding of the molecules to the sites on the surface. Accordingly, we see that a fluid velocity of about $\bar{v}=100$ μm/s already gives a transport rate of about $J\approx 10^{6}$ molecules/s, that is, every 2 s (a time much shorter than typical binding time) the number of molecules hitting the surface reaches $2\times 10^{6}$, corresponding to the resolution of the sensor as we determined earlier.

This analysis allows estimating the typical fluid velocity needed in the sensor, but Equation (1) also reveals power 1/3 dependence between the average fluid velocity $\bar{v}$ and the sensor dynamic. In fact, uniform delivery of bio-analyte to the 9 membranes in the array will require a uniform transport rate that can only be obtained with a good uniformity of fluid velocity on the 1 cm^2^ surface. The main aim of this paper is to point out a strategy to optimize channel design for obtaining good flow rate uniformity across the capture area, while keeping the device compact. Moreover, the reusability of the sensor will also require a clear capacity to efficiently flush the entire volume of the chamber and avoid the existence of “dead zones” where fluid velocity is small.

**Figure 2:**
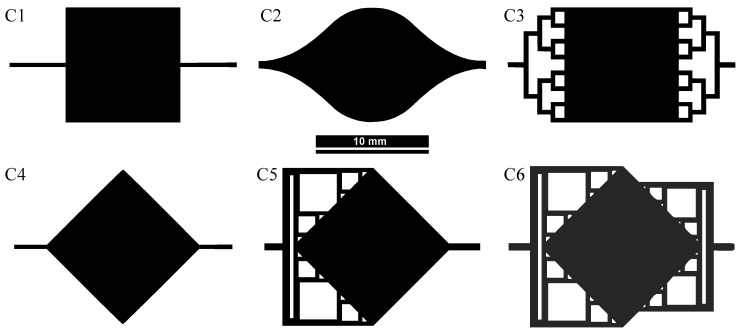
Simulated microfluidic interface topologies with 1 cm^2^ chambers (scale bar is 10 mm).

In order to quantitatively evaluate the velocity uniformity and flush capability in the cavity, we studied the fluid flow with a numerical simulation tool. We study six geometries of microfluidic circuits (Figure 2) exhibiting different topologies of channels in order to compare the distribution of the fluid within the volume of the chamber. The 3 first geometries (C1, C2, C3) are mostly taken from the literature and are used as reference, while the 3 last geometries (C4, C5, C6) are more original to this work. C1 is the simplest configuration with single inlet/outlet configuration, while C2 tries to remedy at the existence of dead zone in the corner of C1 while keeping a single inlet and outlet configuration. C3 is a proposed solution for removing dead zones and getting a uniform flow where a manifold is used at the input and output. The shapes and orientation of the fluidic channels and chambers in C4, C5, and C6 have been adapted for a chip based on anisotropic etching of silicon as will be discussed in Section 3. It should be noted that in these 3 cases, the chamber section varies along the path as the chamber has a diamond shape, and C6 is an attempt to evenly spread the flow at the input and output for better flow homogeneity.

### 2.2. Flow Simulation

As seen in Figure 2, the tree-like architecture that splits the flow into multiple channels can be elaborated with different geometries and size of channels. In these architectures, microchannels have different length and width to obtain the same flow resistance along the input and output path for the different designs. In order to evaluate the velocity field in the cavity, we used the finite element COMSOL multiphysics^®^ software (version 4.2, Comsol Inc., Burlington, NJ, USA) with the microfluidics module. Due to the thickness/length ratio *r* of the microfluidic interface (*r* = 80 μm/1 cm) we used 2D simulation. Simulations of the laminar and incompressible flow in the low depth channel were performed using Navier–Stokes model [18,19]. The fluid was considered to have the same physical properties as water and a pressure difference of 150 mbar was set between the inlet and the outlet of the device. The main simulation results are presented in the following figures.

**Figure 3:**
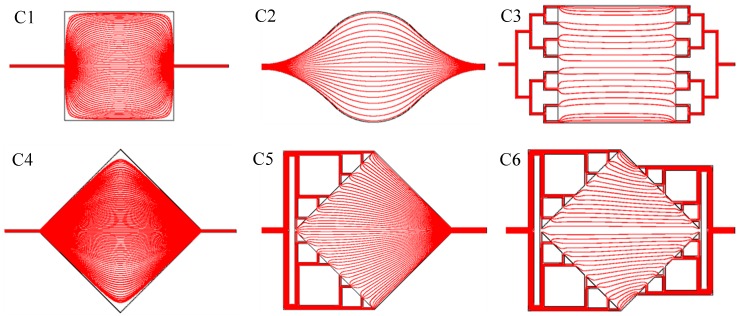
Numerical simulation of flow lines in the microfluidic interfaces of Figure 2.

Figure 3 shows the flow line in the capture area for each interface design. We observe in C1 and in a lesser way in C4, that in the corners, there is little flow as the liquid passing by these distant points will have to follow a very long path and thus present the highest fluidic resistance. These “dead zones” are typical places where we will have difficulty in flushing to rinse the chamber and where eventual bubbles would get trapped. In this respect, C2 substantially improves the flow behavior, with flow line length varying in a smaller range and thus mostly avoiding the “dead zone” apparition. We see also that the manifold structures (C3, C5, C6) improves this aspect too as it evenly spreads the liquid at the chamber inlet and outlet.

**Figure 4:**
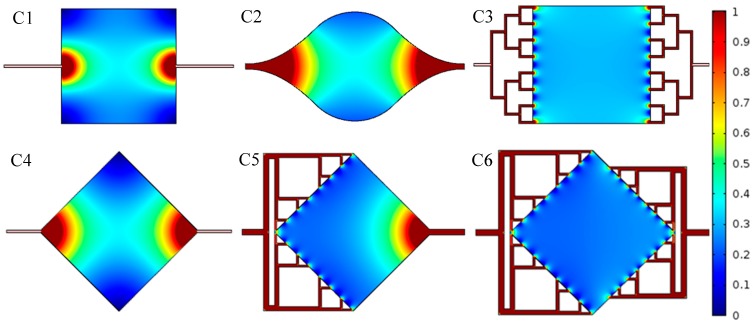
Numerical simulation of the mean velocity of fluid in the microfluidic interfaces of Figure 2 (mm/s) (the scale has been limited to *v* < 1 mm/s to better show the velocity inside the chamber).

Figure 4 gives the mean velocity amplitude in the cavity and channels allowing evaluation of the uniformity of the liquid velocity in the capture area. The velocity profile confirms the existence of “dead zones” in the corners of C1 and C4, with near zero velocity. In fact, by observing these figures, it is clear that the manifold structure at the inlet and outlet of the chamber is the key to providing a uniform velocity distribution of the flow in the chamber. In the C3 and C6 cases, the relative fluctuation of flow velocity in the full chamber reaches 100% (for C6 between 0.25 and 0.5 mm/s) but if we ignore the zone near the manifold channel inlet and consider 98% of the full area, the fluctuation of the flow velocity is only 4%. It is somewhat counter-intuitive that interface C6 is as efficient as C3. Actually, compared to C3 where the chamber section is uniform, the path in the center of the chamber C6 seems much longer than the one at the corner and should present a higher fluidic resistance which in turn would prevent homogenous flow. However, if we look carefully at the manifold structure at the inlet and outlet of C6, we see that they are regularly arranged on the diamond edge, progressively increasing the flow as the section of the chamber increases. We may also note in design C6 that the channel networks are not symmetrical as the inlet and outlet in the chamber are not aligned with each other. Actually, a fully symmetrical manifold architecture gives the worst results because the channels inputs and outputs are too close, leading to less homogenization in the flow.

**Figure 5:**
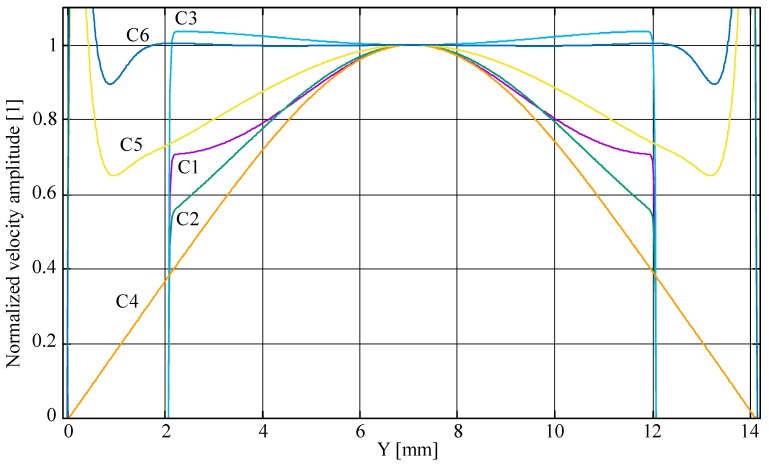
Numerical simulation of the profile of velocity across the middle of the chamber in the microfluidic interfaces of Figure 2 (the velocity is normalized to the velocity at the center of the chamber).

A complementary way to look at the velocity uniformity in the chamber is to compare the fluid velocity in a section in the middle of the chamber, as shown in Figure 5.

C5 and C6 have large velocities at each side of the profile, because the cross-section in the chamber diagonally goes through the input and output channels, which is not the case for the other configurations. We see again in Figure 5 that the C3 and C6 architectures provide better flow uniformity in the chamber, with C6 showing the best uniformity (less than 1% variation of velocity in the central zone outside the input and output channels while C3 has about 4% change in velocity). These two configurations are optimal for the device, simultaneously providing a chamber devoid of “dead zone” and a very uniform flow profile. We will now report on the strategy adopted for the fabrication of the different architectures and compare experimental results with theory.

## 3. Microfabrication

For the fluidic interface, besides its topology, the choice of the material is also decisive. Actually, the fluidic interface needs to successively convey the thiolate solutions, solvents (ethanol, acetone), the deoxidized acid (HCl or H_3_PO_4_ based solutions), the biological liquid, and finally, as the sensor is designed for reusability, the etchant for surface regeneration (H_3_PO_4_, H_2_SO_4_ based baths). The interface will then need to have a high chemical inertness and of course be bio-compatible.

Many materials are commonly used for building fluidic interfaces, such as Polydimethylsiloxane (PDMS), polymers, glass, and silicon, but not all fulfill the stringent requirements needed for a reusable sensor. In Table 1 we have summarized the perceived advantages and drawbacks of different possible fabrication materials. PDMS and polymer are subject to swelling and aging which is not compatible with our reusability objective [20,21]. Glass could be a good choice, however, its coefficient of thermal expansion (16 ppm/K) is large compared to GaAs (6.8 ppm/K) and GaAs chips bonded to glass are known to break easily [22]. Moreover, glass is susceptible to some form of corrosion in H_3_PO_4_ acid, albeit at very slow rate at room temperature [23]. In the end, it appears that silicon fulfills all the material requirements and its micromachining has often been successfully used to fabricate complex bio-microdevices with microfluidic components [16,24]. Dry and wet etching techniques are by far the most common silicon bulk micromachining processes. However, dry etching based on the deep reactive ion etching (DRIE) Bosch process leaves fluorocarbon films on the etched surfaces [25] that are hard to fully remove and could potentially be released during the lifetime of the sensor, thus perturbing the sensing elements. A better and cheaper technique in this case is wet etching with strong base solution (KOH, NaOH) that can be easily rinsed off after fabrication.

### 3.1. Layout of Microfluidic Circuit

In order to keep the device free from potential contamination and at minimal cost, Si structures were made using wet anisotropic chemical etching, a key technology in Microelectromechanical systems (MEMS) fabrication. A substantial amount of research has been conducted to understand the etching mechanism and eventually control the etching shape [26,27,28,29] in a large range of KOH etching conditions. The resultant shape, size, and surface states of the microstructures are restricted by crystallographic properties of silicon. This crystallographic constraint forces the elimination of the rounded C2 architecture for the microfluidic interface. In order to observe the effect of a tree-like architecture that splits the flow into multiple channels, we choose architectures C4, C5, and C6. The formation of rectangular shaped cavities on a (100) Si wafer is well-known in the silicon micromachining process [26,27,28,29]. When the channel edges are aligned along <110> direction, their cross-sections are limited by the flat (100) plane at the bottom and the 54° tilted (111) planes along the two sides of the grooves [28,29]. Considering that the anisotropy of etching is high and (111) is the lowest etching plane, the lateral underetching remains negligible and we can consider that the width of the channel remains equal to the width of the open area of the mask.

**Figure 6:**
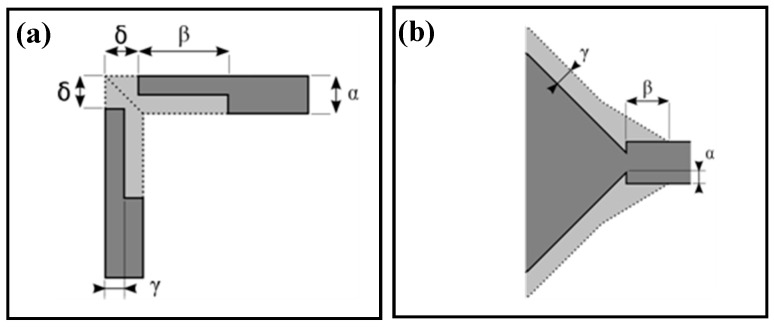
Layout of the compensation structures and definition of parameters: (**a**) layout Q for intersection of perpendicular channels; (**b**) layout I for intersection of channel with cavity. Dotted line and gray area shows the projected etch profile. The dark gray area is the open area of the etching mask.

However, microfluidic channel intersections at the chamber input and output form convex corners. Considerable undercutting occurs at convex corners because, contrary to the concave region, the limiting shape at these corners is bounded by fast etching planes. This results in poor control of the shape and size of the microstructure. Corner erosion leads to rounding of right-angled corners which influences the functionality of the microfluidic structure. The phenomenon depends upon the etchants and their concentrations, etching temperature, and time but most of the literature [27,30,31,32,33] reports that for silicon in KOH solution, {311} or {411} planes emerge as the fast etching planes. Various approaches have been reported in the literature to prevent the undercut at convex corners. The aim of convex corner compensation is to limit the occurrence of the fast etching planes and thereby retard the undercutting until the desired etch depth is achieved. The compensation techniques rely on time-delayed etching of the convex corners through some added structures. Several groups of researchers have proposed a variety of different geometrical structures that are more or less complicated: squares, rectangles, square, and rectangular blocks of various dimensions, triangles [30,31,32,33,34]. The compensation technique used in this experiment is described in [34]. The space required for the compensation is taken in the V-groove length in a simpler way. This method is adapted to any etching depth as long as the V grooves are long enough. The design of the compensation structures and the associated geometrical parameters are given in Figure 6.

The values of the parameters determined using geometrical and etch rates considerations are summarized in Table 2. In this table Q1, Q2, and Q3 are different designs for intersection of perpendicular channels with adjusted compensation parameters to obtain the right sizes for the intersections. I1 gives the values of the parameters for the intersection between the cavity and the channel in the C4 architecture.

For the intersection of the microchannels with the chamber for the C5 and C6 architectures, we placed a gap of 450 μm between the end of channel and the chamber on the mask.

### 3.2. Microfabrication Process

**Figure 7:**
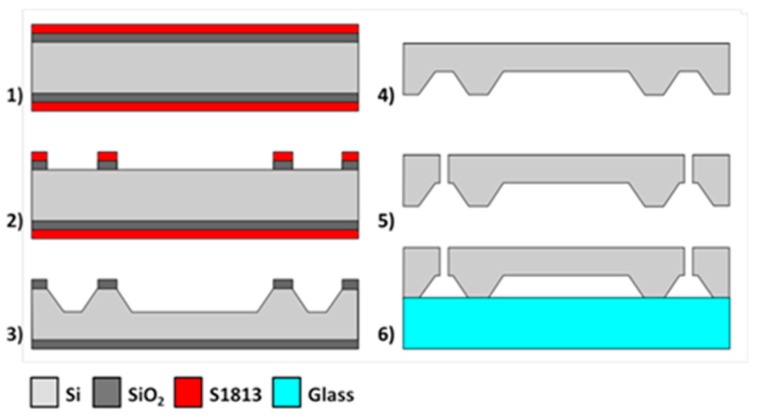
Flow chart of the microfabrication process for the microfluidic interface test.

Figure 7 (steps 1 to 5) describes the process of fabrication for the microfluidic interfaces. We first pattern a 1 μm thick SiO_2_ hard mask grown by thermal oxidation. The KOH etching is then carried out in 35% KOH at 65 °C in a bath that could hold multiple wafers for industrial production. The wafer is then thoroughly cleaned and rinsed to remove any trace of harmful chemical, before the two access holes are opened using laser machining. Alternative processes for the through hole could use dry etching with DRIE, but would pose the residue problem described earlier, while KOH etching of the through holes would give square and large openings not suitable for our fluidic connectors. Moreover, we have also successfully drilled through-holes in the GaAs wafer with laser machining and this could be an option if the fluidic interconnection has to be on the same side than the sensing membranes.

After the etching, the Si fluidic interface is bonded to glass for testing (step 6). Glass is chosen here for image-based study of the fluidic flow properties in the completed structures. In the case of biosensor fabrication, the Si substrate is bonded with the GaAs wafer before the final dicing step.

### 3.3. Micromachined Structures

We used an optical microscope (Mitutoyo FS70, Mitutoyo Corp., Kawasaki, Japan) with a camera (IDS μEye, IDS Imaging Development Systems, Obersulm, Germany) for recording the results of the silicon patterning.

**Figure 8:**
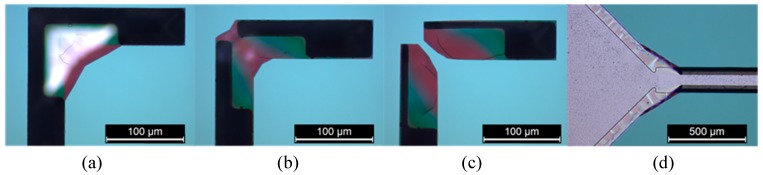
Optical microscopy image of the intersections of channels obtained using several sets of compensation structures, depth etch = 77 μm (**a**) case of set Q1; (**b**) case of set Q2; (**c**) case of set Q3; (**d**) case of set I1.

Figure 8 gives the etched shapes with three sets of geometrical parameters as given in Table 2. An etch depth of 77 μm was reached in the three cases. As expected, the compensation masks limit the corner underetching. As seen in Figure 8c, the channels have not yet merged at the corner, while in case b, the channels are joined and the underetching is limited. The parameters that we retain for the devices are those in Figure 7b corresponding to case Q2, as a slightly rounded corner is rather favorable for the desired flow.

**Figure 9:**
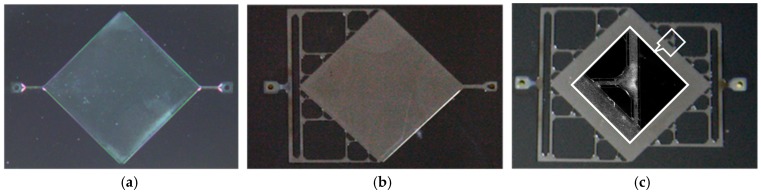
Optical microscope image of the microfluidic circuits after micromachining. Structure (**a**) C4 single input and output; (**b**) C5 single or distributed input and output; (**c**) C6 distributed input and output (central inset showing a zoomed view of the intersection between channels and with the chamber).

Optical images (Figure 9) show the structures after etching during 135 min in KOH solution with the Q2 set of parameters. In the inset of Figure 9c, we observe the development of the {411} planes at the intersection of channels in architecture C5 and C6 and the channel connection with the chamber that have emerged despite the original gap in the mask.

## 4. Experimental Characterization

### 4.1. Experimental Setup

We performed two types of experiment to verify the velocity uniformity and the flush capability: micro-particle velocimetry and colored liquid flow imaging.

**Figure 10:**
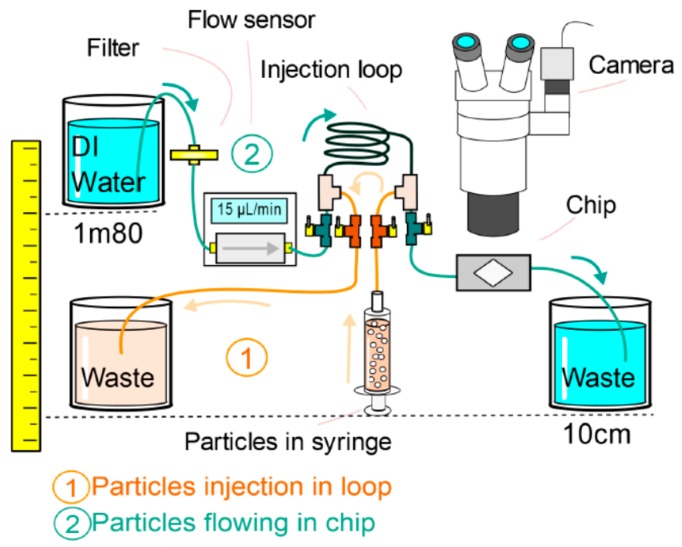
Schematic of the experimental set up used to study velocity flow in the microfluidic interface.

For micro-particle image velocimetry (μPIV) experiments, images were obtained through a binocular with an 80 fps B/W high resolution 2048 × 2048 CCD camera with a high-speed shutter (iDS μEye UI-3370CP-M-GL Rev.2) and a halogen white light source (Schott kl 2500, SCHOTT AG, Mainz, Germany). The binocular (Nikon SMZ800, Nikon Instruments Inc., Tokyo, Japan) was used at ×1 magnification for imaging the full area of the chamber on the camera sensor with a spatial resolution of 5.5 μm/pixel. The high intensity light source and the sensitivity of the camera proved to be decisive for acquiring high enough quality images for PIV post-processing despite the reflective configuration with a textured chamber bottom that created artefacts. We prepared a particle suspension by seeding water with 920 nm monodisperse melanine beads (MF-NB-COOH-S1058, Microparticles GmbH, Berlin, Germany) in a 0.1% *v*/*v* concentration. The chip was first filled with water, then the microparticle suspension was loaded in an injection loop (Figure 10 path 1) before it could be pushed through the chip using clear water (Figure 10 path 2). The use of valves avoids the need for disconnecting tubes, which inevitably leads to introduction of air bubbles to the system. The particles presented a sedimentation velocity estimated at 0.2 μm/s and we tried to keep the distance the particles may sink as they cross the chamber below 10 μm (about 12% of the chamber depth). Accordingly, for the μPIV experiments we used convective flow velocity above 200 μm/s in the chamber, meaning that it takes about 50 s to cross the 10 mm chamber while it sinks by about 10 μm. The velocity was controlled by imposing a hydrostatic pressure difference of about 170 mbar (roughly 1.70 m) between the inlet water tank and the outlet waste reservoir (Figure 10).

For the 3 interface architectures C4, C5, C6, more than 200 image pairs with an interval of 50 ms were taken for PIV processing. Pre-processing and PIV processing of the images have been performed with PyV a Python based custom software [35] developed at FEMTO-ST Institute. During pre-processing, an average background image is subtracted to all instantaneous images, removing reflection and fixed pattern. The selected region of interest (ROI) is the complete chamber, excluding inlet and outlet channels where the flow velocity gradient is too high. Cross-correlation on each image pair gives access to the particle velocity field that is further filtered by removing the spurious vectors where the ratio between the amplitude of the first two peaks of correlation is too small. Eventually, we averaged the 200 instantaneous velocity fields to produce the μPIV images of the fluid velocity in the chamber.

For colored liquid flow imaging, we used a 25 fps color CCD (iDS μEye) and the same light source and binocular as for the μPIV experiment. First, the chamber is filled with clear water and we record the image when red tinted water is injected using the injection loop (Figure 10) for rapid liquid transition without introducing air bubbles.

During all experiments, a flow sensor connected to a PC is placed for continuous recording of the flow rate in the channel, and we kept a constant temperature (22° ± 2°) as it critically influences the physical properties of fluids, especially their viscosities and their surface tension.

### 4.2. Results

**Figure 11:**
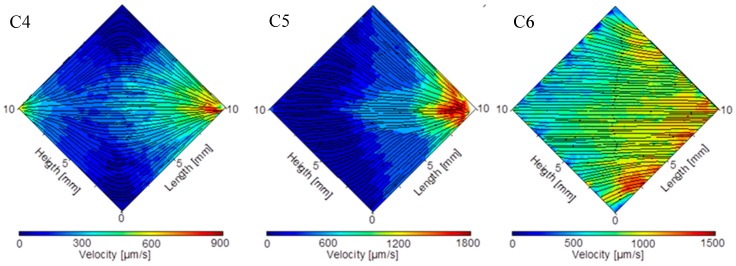
Flow velocity field with streamlines in the chamber measured using micro-particle image velocimetry for the microfluidic interface structure (left) C4, (center) C5, and (right) C6.

Figure 11 shows the measured particle velocity in the large chamber of the 3 interface architectures. Our capacity to achieve simultaneously high resolution and large field of view (1 cm^2^) was important in this experiment, where stitching of smaller images to perform the μPIV would have been complicated because of the lack of features in the large chamber to help stitching. The measurement broadly matches the simulation results presented Figure 4. For example, in architecture C4 (Figure 11 left), we clearly observe the existence of a zone with very low velocity in the north and south corners, and we see a rapid change of velocity as the fluid goes from the right side to the middle of the chamber and then to the left side.

Still, we observe in the 3 structures a gradient of velocity from left to right, that cannot be explained by the fluid behavior only. Actually, in the C4 configuration (and in a smaller measure C6) that has symmetrical inlet and outlet channels, it looks as if more fluid is leaving the chip than is entering it. This can be explained by a combination of multiple factors. First, the image shown excludes a certain zone near the mound of each inlet and outlet channels where the velocity gradient is too high to be measured by μPIV. However, we used a binocular and not a microscope for imaging as it provided a large field with low distortion, but the optical path of each objective is not normal to the surface and is tilted from vertical by an angle of about 6° (convergence angle 12°). The resulting effect is that the mask used to hide the chamber edge is not symmetrically located around the middle of the chamber, with an offset to the right showing more of the channel outlet than inlet. Although we may exclude the effect of inertial focusing due to the small size of the particles [36], sedimentation may be a second factor that creates a gradient of particle velocity by changing the particle distribution along the depth of the chamber. In fact, sedimentation will empty the volume near the glass ceiling of the chamber from its particle, a location where the velocity is the lowest (we assume a parabolic Poiseuille distribution of velocity along the chamber depth). Accordingly, as the μPIV algorithm used computes the instantaneous velocity for particles at any depth in the field, the average across the 200 image pairs will be slightly skewed and we will have an increase of the reported velocity. For the C4 chamber, we used a low flow rate (about 7 μL/min compared to more than 14 μL/min for the experiments with C5 and C6) to visualize the flow close to the input and output and we also notice that its single input/single output configuration forces some particles to follow a path nearly 20 mm long. Accordingly, we estimated the sedimentation depth to more than 16 μm, which is 1/5 of the chamber depth, actually modifying the measured velocity. As discussed in the previous section, for most particles in C5 and C6, the sedimentation depth is less than half that value and its effect is essentially marginal.

**Figure 12:**
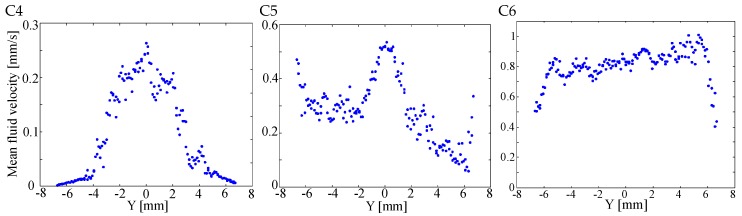
Profile of the mean flow velocity (mm/s) in the middle of the chamber measured using particle image velocimetry for the microfluidic interface configuration (left) C4, (center) C5, and (right) C6.

Another look at the velocity uniformity may be gained by looking at the velocity profile along cross-section in the chamber. We plotted in Figure 12 the velocity measured by μPIV along the longest cross-section located at the vertical diagonal of the chamber (we used a window 400 μm wide around the diagonal to obtain enough data points). This plot compares favorably with the results of the simulation shown in Figure 5.

We observe that the C6 configuration indeed provides the best flow uniformity that can be estimated here at ±10% (excluding 1 mm at the side of the chamber). On the other hand, the flow velocity uniformity may be estimated at ±70% for the C5 configuration and at ±100% for the C4 structure. The remaining gradient in the C6 architecture is due to non-uniformity in the inlet channel width. Actually, the simulation results shown in Figure 3 and Figure 4 are based on identical channels at the inlet and outlet manifolds. During fabrication, small variations in channel widths are inevitably introduced that break the ideal symmetry of the structure, inducing small velocity differences at the mound of each channel in the chamber. In fact, we suppose that the oscillations that can be seen in the velocity profile (we may roughly count 8 minima across the chamber diagonal) are directly linked to the velocity differences at the channel mounds. It is expected that a better control of the fabrication process will allow further improvement of the uniformity.

**Figure 13:**
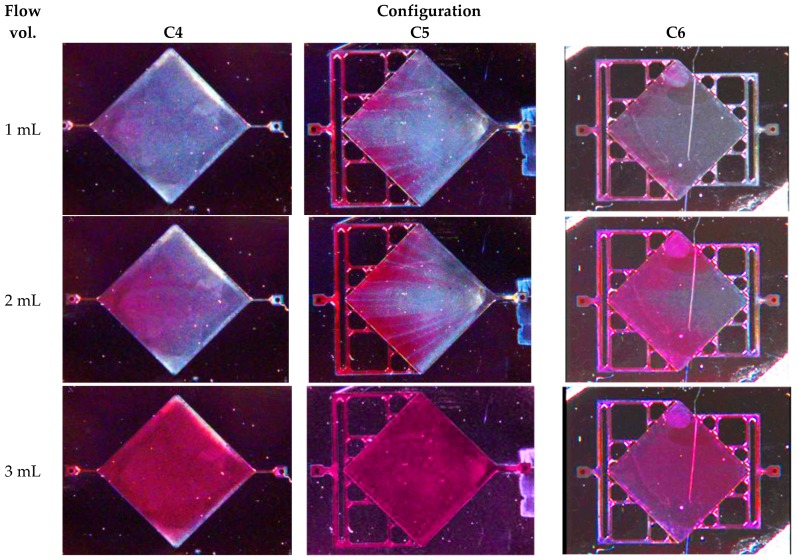
Flush sequence (shown as a function of liquid volume to facilitate comparison) in configurations (C4), (C5), and (C6) (red tinted water is entering from the left) (see movie for C5 configuration in online additional materials).

We also studied the effect of flushing the chamber, particularly for studying the process of regeneration of the sensor where multiple chemicals will be passed over the membrane surface to restore its capture properties. Figure 13 presents the result of using the colored liquid flow experiment with the different interface configurations.

It is interesting to note that the filling of the cavity reveals streaks in the liquid (particularly for configuration C5) that closely match the stream lines for the three selected architectures as simulated in Figure 3. Actually, as the liquid flows inside the chamber, we have the formation of flow cells separated by thin interfacial zones that start roughly at the mid-point between inlets and where the velocity is lower. Those slower interfacial zones, which were already observed as velocity oscillations in Figure 12, retain the blue liquid longer, revealing the flow line in the chamber when the red liquid enters. Additionally, the observation of the evolution of the frontier between the red and the blue zones as a function of time shows the shape of the flow front an information not given by the steady state numerical analysis. We see that the flow front is much more uniform in the C6 architecture than with the C5 architecture, even if the middle of the chamber is flushed slightly later, presumably because of etching non-uniformities in the channel widths. The second row clearly highlights the drawbacks of the C3 architecture with “dead zone” at the north and south corners and a non-uniform flow front.

Some supplementary tests were also performed to evaluate the behavior of the microfluidic interface during filling at the first liquid injection when the chamber is still filled with air. These tests help to appreciate the tolerance of the design with respect to bubble creation, an often-encountered issue in microfluidics. In this case, the best performance is obtained with the C5 interface, with the C4 interface performing worse and the C6 interface having bubble issues from time to time. Filling C5 from its multiple inlet allows a regular filling of the chamber without trapping air that is easily evacuated through the single outlet channels (see movie in online additional materials). The interface of C6 will need to be filled slowly using capillary pumping for best results, while the C3 configuration will need patience to avoid any air droplets becoming trapped in the corners of the chamber.

## 5. Conclusions

The fabrication of a successful biological sensor requires merging a large number of technologies that should be thoroughly evaluated. We have presented results of fluidic simulation and experiments conducted to study the flow in a large-size biosensor for specific molecule detection. We improved the uniformity of flow in the capture area and provided a robust and cheap structure suitable for a reusable sensor. Future work will be conducted with a functionalized surface to quantify the homogeneity of molecule capture.

We note that the tests have been performed with a glass wafer instead of the piezoelectric material (GaAs), which could potentially change the behavior of the flow. However, the fluid in the channels is in contact with silicon on three sides and only the material on the top of the channel may change, which should not modify too strongly the behavior observed in this work. Ultimately, we will need to demonstrate the efficiency of this architecture using the complete acoustic sensor.

These results may be applied to immunoassays in microfluidics and to all applications requiring a high capture area in a compact footprint, including the optimization of commercial products.

## Figures and Tables

**Table 1:** Comparison of materials for the reusable biosensor microfluidic interface.

Material	Material Cost	Compat. with GaAs	Fabrication Cost	Resistance to Aging	Resistance to Regen.	Biocompat.
PDMS	low	medium	low	low	medium	high
Polymer	low	medium	low	low	low	medium
GaAs	high	high	medium	high	high	medium
Glass	medium	low	high	high	medium	high
Silicon dry	medium	medium	high	high	high	medium
Silicon wet	medium	medium	medium	high	high	high

**Table 2:** Values (in μm) of geometrical parameters for the compensation structures.

Set	α	β	γ	δ
Q1	100	170	50	0
Q2	100	170	50	50
Q3	100	210	25	50
I1	50	200	75	-

## Supplementary Materials

Supplementary File 1
